# Operational Performance of a Compact Proton Therapy System: A 5-Year Experience

**DOI:** 10.14338/IJPT-21-00033.1

**Published:** 2022-07-01

**Authors:** Omar A. Zeidan, Ethan Pepmiller, Twyla Willoughby, Zhiqiu Li, James Burkavage, Brian Harper, Michael Fraser, Katie Moffatt, Sanford L. Meeks, Naren Ramakrishna

**Affiliations:** Department of Radiation Oncology, Orlando Health Cancer Institute, Orlando, FL, USA

**Keywords:** proton therapy, compact systems, operational performance

## Abstract

**Purpose:**

We present an analysis of various operational metrics for a novel compact proton therapy system, including clinical case mix, subsystems utilization, and quality assurance trends in beam delivery parameters over a period of 5 years.

**Materials and Methods:**

Patient-specific data from a total of 850 patients (25,567 fractions) have been collected and analyzed. The patient mix include a variety of simple, intermediate, and complex cases. Beam-specific delivery parameters for a total of 3585 beams were analyzed. In-room imaging system usage for off-line adaptive purpose is reported. We also report key machine performances metrics based on routine quality assurance in addition to uptime.

**Results:**

Our analysis shows that system subcomponents including gantry and patient positioning system have maintained a tight mechanical tolerance over the 5-year period. Various beam parameters were all within acceptable tolerances with no clear trends. Utilization frequency histograms of gantry and patient positioning system show that only a small fraction of all available angles was used for patient deliveries with cardinal angels as the most usable. Similarly, beam-specific metrics, such as range, modulation, and air gaps, were clustered unevenly over the available range indicating that this compact system was more than capable to treat the complex variety of tumors of our patient mix.

**Conclusion:**

Our data show that this compact system is versatile, robust, and capable of delivering complex treatments like a large full-gantry system. Utilization data show that a fraction of all subcomponents range of angular motion has been used. Compilation of beam-specific metrics, such as range and modulation, show uneven distributions with specific clustering over the entire usable range. Our findings could be used to further optimize the performance and cost-effectiveness of future compact proton systems.

## Introduction

The utilization of compact proton therapy (PT) systems has been steadily increasing for the past 10 years. Despite efforts to miniaturize PT systems, cost remains a major obstacle for wider implementation of PT [1]. In addition, PT reimbursement rates remain uncertain in the era of accountable care [2] and the implementation of the Radiation Oncology Alternative Payment Model [3]. For these reasons, technologic innovations are likely the most viable solution for cost-effective compact system solutions while maintaining their clinical robustness compared with traditional full-gantry systems. The key to gauge innovation success relies on user feedback of clinical and operational utilization of these systems. This utilization data would be most useful to manufacturers if they span the use of the system over an extended period and for many indications with varying complexity.

Several studies reported on various PT systems performance with emphasis on delivery times [4–6]. However, these studies reported on technical data for large multi-room centers with full-gantry rooms. These centers operate differently than single-room compact systems. First, treatment rooms in a large center share a beam line originating from a single-beam source or accelerator. Thus, in-room workflow is mainly dictated by the serial nature of beam availability from the shared beam source. In comparison, each single-room or compact system has, by design, its own dedicated beam source. Second, the in-room workflow of a full-gantry system lends itself to coplanar beam delivery with less frequent robotic movements compared with a compact system with limited gantry range.

Here, we report on clinical and technical operational data of a first-generation Mevion S250 passive scattering compact system (Mevion Medical Systems, Littleton, Massachusetts). Beam-specific data on 850 patients treated within the first 5 years of operation were collected and analyzed. This compact system's unique design features a gantry-mounted superconducting accelerator without a beamline between the accelerator port and treatment room nozzle. The concept of gantry-mounted accelerator was first devised by Blosser in 1989 [7]. The rotation of the accelerator around isocenter and against gravity poses some challenges in maintaining tight beam specs with gantry angle due to high mechanical stress on the accelerator and field shaping system (FSS) components. In contrast, all other commercially available compact PT systems employ a stationary, horizontally mounted accelerator with a beam line between the accelerator and nozzle.

## Materials and Methods

### System Description

#### General

The first patient was treated on April 6, 2016. At the time, our facility was the fourth worldwide to operate this type of compact system. The facility is part of Orlando Health Cancer Institute downtown campus and connected to the main radiation oncology department. The 3-story vault is nearly 28 ft high with the treatment room as the middle level. This height allows for the outer gantry structure to swing around the treatment room over a span of 190°. The outer gantry has 2 major hardware components: (1) a cylindrical housing that encapsulates the vertically mounted superconducting synchrocyclotron with access points on either side of the accelerator. Thus, the plane of proton beam within the accelerator is the same as the gantry rotation plane. (2) Two counterweights mounted at opposite ends of the cylindrical housing forming a U-shaped gantry structure for precise and balanced rotational motion. The entire gantry structure weighs nearly 225 000 lbs. The inner dimensions of the treatment room are approximately 18 × 21 ft with an x-ray booth measuring 5.5 × 7 ft at one corner the room facing the maze entrance. The x-ray booth is used to acquire daily kilovolt-to-kilovolt alignment images in addition to 3-dimensional (3D) images from a mobile computed tomography (CT) unit.

#### In-room hardware

**Figure 1.**
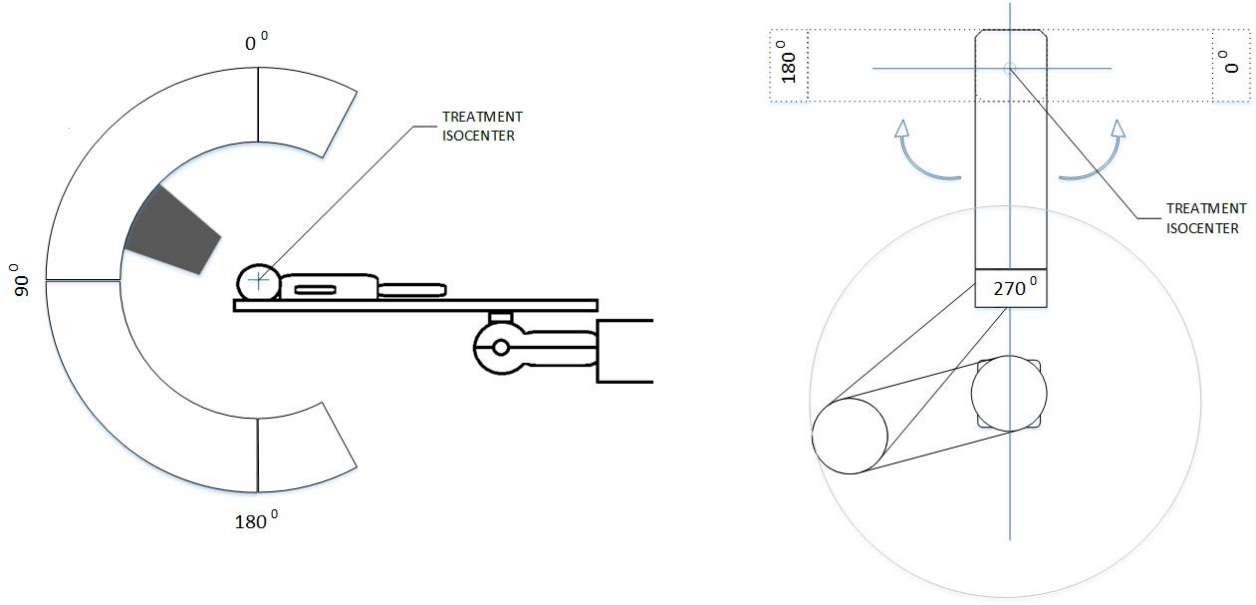
Schematics of major components insider the treatment room. The inner gantry side view (left) and an overhead view robotic couch (right) with corresponding angle conventions.

The major hardware components of the treatment room are the C-shaped inner gantry (CIG) and the patient positioning system (PPS). A simple graphic representation of the CIG and PPS and their angle convention is shown in **Figure 1.** It is worth mentioning that the robot can rotate by additional 45° past the 0° and 180° positions to couch position 45° and 135°, respectively. Similarly, the CIG can move by additional 5° past the 0° and 180° to 355° (clockwise) and 185° (counterclockwise), respectively. Upon request of a beam, the control system enables CIG motion so the therapist can move the snout through a hand pendant controller to the planned CIG angle. This is followed by motion of the outer gantry. Final alignment of both gantries is achieved through optical sensors. The CIG structure houses the nozzle components and snout. For operational simplicity, the system offers only 2 size applicators that attach to the snout, accommodating maximum diameter openings of 25 and 14 cm for the large and small applicators, respectively. The PPS is mainly composed of a carbon fiber couch top mounted on a 6°-of-freedom robot (KUKA Roboter, GmbH, Augsburg, Germany). The robot and couch top can support a maximum load of 360 lbs. The PPS can execute submillimeter shifts for translational motions and 0.1° motions around the 3 rotational axes. It is worth mentioning that the use of a surface-based monitoring system is challenging due to limited field of view of the patient surface insider the room. This is mainly due to relatively low ceiling clearance and the blocking of the camera view of the patient at setup position when x-ray panels are deployed. In addition, the internal dosimetry system is not designed to interface with third-party motion management solutions to provide active beam gating capabilities.

#### Passive scattering system

The passive scattering system was designed with the goal of providing the following: (1) 2 field sizes measuring 14 cm × 14 cm and 25 × 25 cm at isocenter; (2) 3 field types that reflect the depth and size of tumors, including large (options 1–21) for breast-like treatments, deep (options 13–17) for prostate-type treatments, and small (options 18–24) for intracranial-type tumors; (3) fine adjustment capability of 1 mm (0.1 g/cm^2^) for beam range and modulation, and (4) dose uniformity of ±3% throughout 80% a cone volume centered at iso with a 25-cm diameter. To achieve these specifications, each element of the FSS is serially configured in a specific sequence to deliver the desired option. These elements include first scatterer, range absorbers, second scatterer and 14 different modulation wheels. The 24 available options provide clinically deliverable beams with a range span of 5.0 to 32.0 gm/cm^2^ and a modulation span of 2.0 to 20.0 gm/cm^2^. Regardless of the option, these elements are arranged within a nominal 200-cm source-axis distance.

### Data Tracking

Technical beam data of patient treatments were compiled by our proton team since the first day of going live. The data were extracted from patient treatment plans and the oncology information system (OIS) and then entered manually in various spreadsheets. The percentage of cancelled proton fractions out of scheduled fractions was tracked as it represents a clinically meaningful surrogate for overall system uptime. Fractions that are not scheduled in OIS due to major predetermined routine maintenance are not included as cancelled fraction in our analysis. This is a different approach than using system availability metrics that track treatment room availability for scheduled use such as patient treatment, research, and development [8]. It is worth mentioning that some proton patients receive intensity-modulated radiotherapy fractions on the same day their proton fractions are cancelled depending on linac-based availability, a physician-approved, intensity-modulated radiotherapy backup plan, and timing of the cancellation. For our system, there were 3 major causes for fractions cancellations, including (1) unexpected hardware-related issues, (2) environmental and institution-related issues, such as power outage due to severe weather or disruption of ancillary services, such as chilled water supply, or (3) planned or scheduled preventive maintenance, such as cryogenics component replacements.

### Routine Quality Assurance

Our daily, monthly, and annual quality assurance (QA) tasks and corresponding tolerances follow closely the recommendations of TG 224 [9]. A comprehensive reporting on all aspects of QA for this system is not feasible within the content and format of this manuscript. Therefore, we opted to report on QA parameters over time for the most frequently used components of this system. Because of its unique design compared with a full-gantry system, we report on beam parameters with CIG and PPS angles and their congruencies with the imaging isocenter. These parameters were measured on a monthly and annual basis and include (1) relative output with CIG angle, (2) symmetry and flatness with CIG angle, (3) congruency of CIG radiation isocenter with imaging isocenter, (4) congruency of robotic angle mechanical isocenter with imaging isocenter, and (5) various range and modulation values.

Relative output of the standard beam (range = 15 gm/cm^2^, modulation = 10 gm/cm^2^) with CIG angle is measured using a farmer-type chamber that is inserted at mid-modulation depth in an acrylic phantom and attached to the snout. Symmetry and flatness of the reference beam were measured for different gantry angles using a STARCHEK 2D array (PTW, Freiburg, Germany) following the TG 224 recommendation. The array was sandwiched between slabs of solid water at mid-modulation depth of the standard beam and held in place at the end of the couch by a mounting bracket that allows the solid water and detector stack to be positioned in 22.5° increments. This arrangement allowed for measuring symmetry and flatness of the standard beam for 9 equally separated CIG angles.

To measure the congruency of the CIG radiation and PPS angle isocenters with imaging isocenter, the Winston-Lutz technique was implemented [10]. A simple cube phantom with an implanted central BB was used to align the phantom to imaging isocenter. Radiochromic film pieces were then placed on the cube surfaces that face the 3 CIG cardinal angles to be measured. The films were then irradiated using a circular aperture. Analysis of the film provided vector difference in millimeters between the imaging isocenter (BB center) and circular radiation pattern center (radiation isocenter).

The congruency of imaging and robot rotational isocenter (mechanical isocenter) was measured using an imaging phantom fixated at the end of a tall couch-indexed stand to allow for kilovolt-to-kilovolt imaging at all possible couch angles. Once the BB is aligned to imaging isocenter at setup position, the robot is moved to different angles and imaging is repeated at each angle and the vector difference between the imaging and BB isocenter is recorded for that angle.

Measurements of range and modulation with submillimeter precision are challenging for noncardinal angles. Therefore, we report on these 2 important beam parameters for a specific set of range and modulation values using PTW's MP3-M 3D water tank with a PTW Markus chamber (TN34045) at 0° gantry position. These measurements are repeated on an annual basis to ensure the physical integrity and accurate positioning of FSS components over time.

### Imaging Systems

Daily imaging is performed with fixed orthogonal x-ray sources and 2 movable detector panels that provide a posteroanterior and lateral images at setup position (couch = 270°). In addition, a mobile big-bore CT unit provides 3D imaging capabilities. The AIRO mobile CT system (Stryker, Kalamazoo, Michigan) was the first of its kind to be installed in any PT system for routine in-room CT imaging. The AIRO was used on 290 patients since its commissioning in December of 2016. A total of 861 in-room scans were performed with an average of 3 scans per patient through their course of treatment. The scans were performed on a selected group of patients per physician request based on their clinical indication. The AIRO was used for scanning several disease sites including thorax, breast, and head and neck (H&N). These 3 sites combined accounted for nearly 70% of all scans. The remaining sites include brain, abdomen, and pelvis. When not in use, the system is stowed in the maze just outside the treatment room. The characterization of the system and its use for offline adaptive workflow is described in previous work by our group [11, 12].

## Results

### Treatment and Delivery Statistics

A total of 25,567 fractions were delivered averaging nearly 419 fractions/month. The number of monthly fractions continued to increase during the first 2 years and averaged 472 per month for the past 3 years. **Table 1** lists the main disease sites that were treated with corresponding planned beam-related statistics per site. Most beams (44%) were delivered for central nervous system (CNS) cases. Breast followed by CNS and H&N, required the most beams per plan. The number of pediatric patients was 153 (17.9% of all cases) with 42 cases requiring anesthesia (27.5%). A total of 34 CSI cases were treated of which 26 were pediatrics (76.5%). Certain disease sites, such as breast and prostate, use a single applicator while the rest of the sites use a combination of 2 applicators based on the initial and boost volumes. The small applicator was used for 64.7% of all beams. In-room time for imaging and beam delivery varies per disease site. The time from the start of imaging at site setup to end of beam delivery based on 10 patients per disease site and averaged over 5 fractions per patient is 12.5, 17.2, and 23.1 minutes for prostate, H&N, and lung, respectively. The scheduled treatment times in OIS for these sites is typically fixed at 15 minutes for prostates and 30 minutes for H&N and lung to account for various delays.

**Table 1.** A breakdown of disease site, patients (N = 850), and number of beams used per site.

**Site**	**Patients, n (%)**	**Beams, n (%)**	**Beams**	**Applicator**
Breast	83 (9.8)	397 (11.6)	5	Large
CNS	264 (31.0)	1501 (43.7)	4^a^	Small^a^
H&N	107 (12.6)	470 (13.7)	4	Mixed
Prostate	246 (28.9)	547 (15.9)	2	Small
Thorax	100 (11.8)	343 (10.0)	3	Mixed
Pelvis or Abdomen	46 (5.4)	158 (4.6)	3	Mixed
Extremities	5 (0.5)	15 (0.4)	3	Large

**Abbreviations:** CNS, central nervous system; H&N, head and neck.

a Excluding CSI.

**Figure 2.**
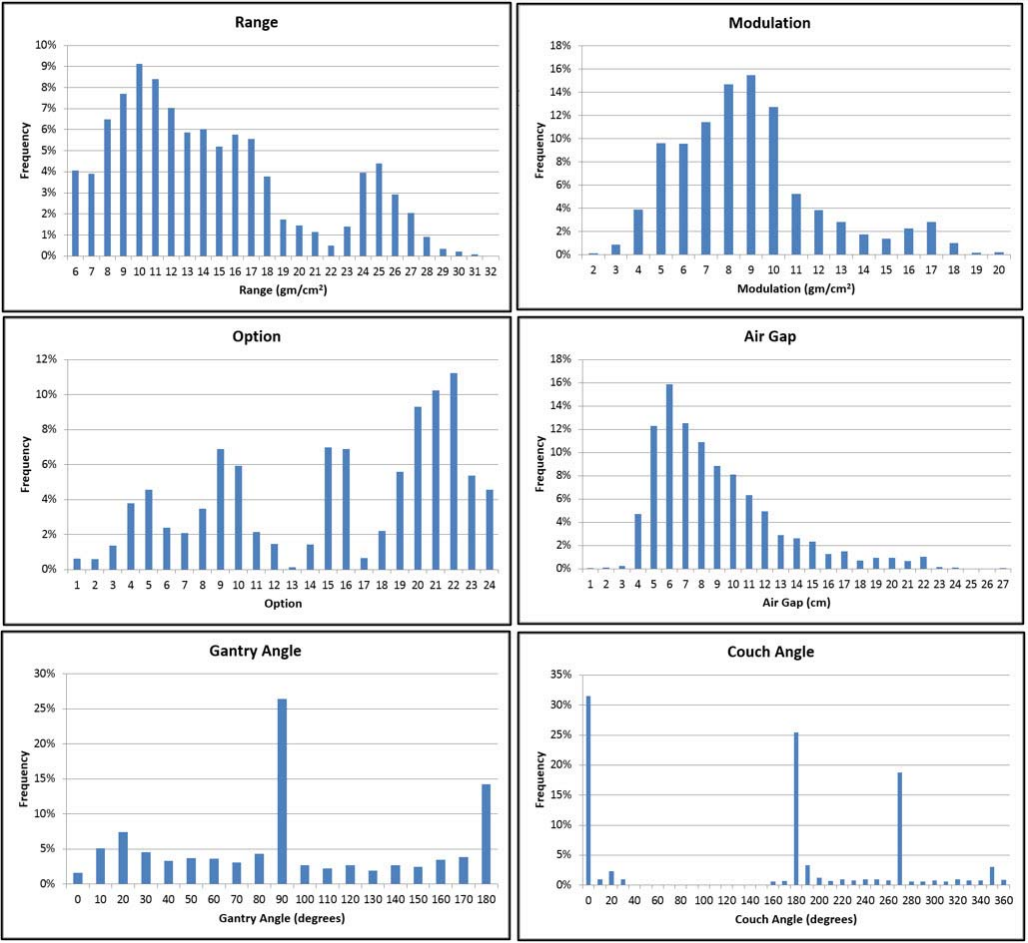
Histograms of clinical beam delivery parameters.

**Figure 2** shows various beam-related utilization histograms including range, modulation, options, air gaps, gantry angles, and couch angles. In each category's histogram, excluding options which are numbered 1 through 24, a bin represents a range of values that counts toward that bin and extends from any value beyond the last bin's numeric value up to the actual numeric value of the bin. For example, the gantry angle bin of 80° is the sum of all angles between 70.1° to 80°. Similarly, the modulation bin of 11 gm/cm^2^ is the sum of all modulation values between 10.1 and 11 gm/cm^2^.

#### Range, modulation, and options

**Figure 2** shows histograms of range, modulation, and option used for all delivered beams (n = 3583) during the 5-year period. The range histogram shows 2 distinct distributions peaking at around 10 and 25 gm/cm^2^. The distribution around the deepest range values is mostly dominated by prostate beams. The most widely used range values fall within the 5 to 11 gm/cm^2^ (78%). Most beams used a range less than 27 gm/cm^2^ (96.4%). For the modulation histogram, the histogram peaks in the 7 to 10 gm/cm^2^ range with most modulation values within 4 to 17 gm/cm^2^ (97.6%). This modulation span is nearly 72% of the total clinically available modulation span provided by the system.

Overall, there is no clear correlation observed between the modulation and range histograms. This can be attributed to the fact that we treated a wide variety of tumors at varying depths and sizes. For example, prostates tend to have deep ranges and relatively tight modulation windows (8–10 gm/cm^2^) while breast cases tend to have comparable range and modulation values. **Figure 2** shows uneven distribution of option utilization that reflects our complex patient case mix. Of note, 7 of 24 available options—1, 2, 3, 12, 13, 14, and 17—were used the least (<5% combined). In essence, the system was sufficiently equipped to treat 95% of all our patient mix using only 17 options (71% of all options).

#### Air gaps

We report the air gap for a given beam as the in-air separation between the surface of the compensator and patient surface along the central axis of the beam. The air gap is calculated per beam manually based on the values of maximum compensator thickness, snout position, and isocenter location in Eclipse. It is an important parameter to quantify because it is closely correlated to beam lateral penumbra at depth [13]. The smaller the air gap, the sharper the penumbra, which is beneficial in cases for where target and organs at risk are abutted. Owing to their relatively large physics size, large applicators tend to have larger air gaps than smaller applicators to avoid collision scenarios with patient and couch hardware. **Figure 2** shows that most air gaps (85%) fall in the range of 4 to 12 cm, which is only 23% of the dynamic range available for the snout motion (7–42 cm).

#### CIG angles

Our utilization data show that most beam angles except for cardinal angle bins 90° and 180° were used relatively uniformly at a rate of nearly 5% or less. The 90° angle was the most used (26.4%) followed by 180° angle (14.2%). The 0° cardinal angle was the least used of all angles at 1.6%. A total of 11 beams (0.3% of all beams) have used the extended range 0° to 355° (clockwise) and 180° to 185° (counterclockwise).

#### Couch angles

The couch histogram in **Figure 2.** shows the following 3 dominant cardinal angle bins: 0° (31.5%), 180° (25.4%), and 270° (18.8%). Most other angles have nearly 3% or less utilization rate. Only 5.6% of all beams used the couch in the extended 90° span (0°–45° clockwise and 180°–135° counterclockwise). The rest of the beams were delivered in the 180°-range span shown in **Figure 1** out of the total allowable 270° span.

### Quality Assurance

Standard beam relative output was measured for f5 gantry angles as shown in **Table 2.** Each output value is normalized to gantry 90° output position. All values are within ±1% with a maximum variation of 0.7%. The data show no clear trend over time. Flatness and symmetry values were measured every year for 9 gantry angles starting the second year. The device that allows for measuring flatness and symmetry was not available during the first year. Each data point is the average of the inline and crossline values. There is no clear trend in the values over time and both flatness and symmetry fall within the recommended values in TG 224 of ±2%.

**Table 2.** QA parameters for various CIG and couch angles are relative to baseline (Y1) except for flatness and symmetry.

**CIG/couch angle**	**Relative output, %**				**Flatness, %**				**Symmetry, %**				**WL, mm**				**I/M congruency, mm**			
	**Y2**	**Y3**	**Y4**	**Y5**	**Y2**	**Y3**	**Y4**	**Y5**	**Y2**	**Y3**	**Y4**	**Y5**	**Y2**	**Y3**	**Y4**	**Y5**	**Y2**	**Y3**	**Y4**	**Y5**
0	0.0	–0.1	0.2	0.4	0.9	1.0	1.0	1.3	1.3	1.0	1.1	1.5	–0.4	–0.4	0.1	–0.1	0.19	–0.08	–0.02	0.26
22.5	—	—	—	—	0.9	1.0	1.3	1.1	1.0	1.2	1.8	1.2	—	—	—	—	—	—	—	—
45/315	0.0	0.2	0.3	0.6	1.0	0.9	1.3	1.1	1.1	1.0	1.7	1.2	—	—	—	—	–0.17	–0.23	–0.18	–0.13
67.5	—	—	—	—	1.0	1.0	1.5	1.1	0.9	1.2	2.1	1.4	—	—	—	—	—	—	—	—
90/270	0.0	0.0	0.0	0.0	1.0	0.9	1.4	1.1	1.2	1.0	1.6	1.1	–0.2	–0.1	0.0	–0.2	0.0	0.0	0.0	0.0
112.5	—	—	—	—	1.1	1.0	1.4	1.4	1.3	1.0	1.6	1.5	—	—	—	—	—	—	—	—
135/225	0.0	–0.1	0.3	–0.2	1.1	1.0	1.5	1.2	1.5	1.2	1.7	1.7	—	—	—	—	–0.33	–0.34	–0.2	–0.17
157.5	—	—	—	—	1.1	1.1	1.3	1.3	1.6	1.4	1.6	1.9	—	—	—	—	—	—	—	—
180	0.0	0.2	0.7	–0.1	1.3	1.0	1.5	1.2	1.8	1.4	1.7	1.6	–0.4	–0.3	0.0	0.0	–0.3	–0.29	–0.03	–0.01

**Abbreviations:** CIG, C-shaped inner gantry; WL, Winston-Lutz test; I/M, imaging and mechanical (robotic couch).

Note: Dashes indicate xxxx.

The congruency of the CIG radiation isocenter and imaging isocenter is a measure of the integrity of the CIG mechanical alignment over time. Each Winston-Lutz test value in **Table 2** represents the relative difference to year 1 value in millimeters. All values are within ±0.5 mm from year 1 value indicating that the radiation and imaging isocenters have maintained a tight congruency over time despite the considerable snout weight and frequent motion within the CIG.

Congruency of the imaging panel isocenter and robot mechanical isocenter for 5 different robot angle positions are shown in **Table 2**. For each couch position the vector distance in millimeters between the center of the BB and imaging panel isocenter is measured. Each value in **Table 2** represents the congruency relative to year 1. The data show no clear trend over time with a maximum deviation of 0.33 mm.

**Table 3** lists specific range and modulation relative values compared with year 1 for all options. All range values are within ±1 mm except for 2 in option 3 where they are above the tolerance by 0.1 mm. All modulation values, with no exception, are within the ±2%/±2-mm tolerance in TG 224. There is no clear systematic trend in any option. This indicates that the FSS elements, despite their constant motion against gravity, have maintained a tight mechanical alignment over the 5-year period.

**Table 3.** Range and modulation variations per year relative to baseline (Y1).

**Field**	**O**	**R, mm**	**M, mm**	**Range in water, mm**				**Modulation in water, mm**				
				**Y2**	**Y3**	**Y4**	**Y5**	**Y1**	**Y2**	**Y3**	**Y4**	**Y5**
Large	1	250	100	–0.2	0.2	0.2	0.5	1.0	2.2	2.4	1.1	2.0
	2	225	100	–0.1	0.4	0.0	0.3	–1.5	1.2	2.2	1.5	–0.6
	3	208	150	0.5	1.1	0.8	1.1	–0.3	–1.6	–1.0	1.9	–1.8
	4	187	100	–0.8	0.3	0.2	0.5	1.7	2.0	1.6	1.7	1.2
	5	167	100	0.0	0.3	0.5	0.8	1.1	1.8	0.4	1.7	1.4
	6	148	100	0.5	0.3	0.3	0.6	–0.9	-1.3	–2.3	–0.6	0.6
	7	131	80	–0.1	0.4	0.1	0.5	–0.5	1.4	–1.7	0.6	1.5
	8	114	80	0.3	0.3	0.2	0.7	–0.9	–1.4	–2.0	0.4	–0.6
	9	99	50	0.6	0.3	0.3	0.3	0.6	–0.7	–0.3	0.2	0.5
	10	85	50	0.6	0.3	0.5	0.6	0.7	0.6	0.2	1.1	1.5
	11	72	50	0.0	0.2	0.4	0.6	–1.0	0.8	–0.5	–1.4	1.2
	12	60	40	0.0	0.1	0.3	0.7	0.4	0.8	0.7	1.2	–0.3
Deep	13	320	100	0.0	0.7	0.7	0.7	–0.3	–0.3	–0.4	–0.8	–0.6
	14	295	100	0.3	0.9	0.8	0.6	0.3	–0.6	–0.3	–0.7	1.5
	15	270	100	0.1	0.6	0.7	0.8	–0.4	0.0	–0.5	–1.0	2.4
	16	245	100	0.3	0.5	0.1	0.7	1.8	–0.1	0.6	–0.9	–1.9
	17	210	100	0.0	0.4	0.5	1.0	2.2	2.2	1.5	–0.8	2.1
Small	18	200	100	–0.2	0.4	–0.1	0.4	1.7	1.2	–0.3	–0.5	1.5
	19	177	100	0.4	0.8	0.1	0.3	2.4	1.5	0.6	1.2	1.9
	20	150	100	0.3	0.8	–0.1	–0.2	0.6	–2.2	–0.6	0.5	1.5
	21	130	100	–0.2	0.5	–0.3	0.6	–0.3	0.5	-0.2	–0.4	–1.7
	22	110	100	–0.4	0.3	–0.7	–0.1	2.2	1.5	–1.7	–0.3	–1.2
	23	85	70	0.3	0.9	–0.1	0.9	2.3	2.1	1.0	1.0	1.9
	24	65	50	0.0	0.5	–0.6	0.2	1.9	–0.4	0.0	0.5	1.9

**Abbreviations:** O, option; R, range (as measured in water); M, modulation (as measure in water).

### In-room CT Imaging

Of 287 patients that were scanned with the AIRO system, only 18 patients (6.3%) had a plan revision during their course of treatment. Nearly 80% of all scans performed were for lung, breast, and H&N cases. Plan revisions or adaptations were triggered by either an observed change in target or surrounding tissue upon examination of the AIRO image set fusion to planning CT set. Some revisions involved a simple change in range and modulation values for one or more beams. In these cases, patient devices (apertures and compensators) remained the same as the original plan if target coverage was restored to the original plan.

### Uptime

After the one-half hour daily warm-up procedure, which is performed by a therapist, our treatments start promptly at 7 am. For the past 2 years, treatments finished around 8 pm in nearly 62% of all clinical days. Nearly 3.8% of all scheduled fractions in the 5-year period were cancelled due to the combined effect of 3 main causes listed earlier. Hardware-related issues were responsible for 2.1% of all cancellations. Nearly 42% of all months had zero fractions cancelled. By comparison, the average fraction cancellation rate for all 9 external beam modalities in our department was 1.7% for calendar years 2019 and 2020.

## Discussion

To our knowledge, this work presents the largest technical and clinical data ever reported on the utilization of a novel compact PT system. The 2 main aims of this work are as follows: first, to evaluate performance metrics for the most widely used system components through temporal monitoring of relevant QA tasks. This is necessary to determine the robustness of the system and its subcomponents when operated at full capacity over an extended period. Second, to present data analysis on clinical use of the system subcomponents to understand the rate of usability of each subcomponent. This is necessary to determine if any of the subcomponents and their dynamic functionality range are well suited for treating a wide range of indications.

Our data show that we have managed to treat a variety of indications with complex deliveries like a full-gantry system [14]. The system has an overall reliability that is comparable to conventional external beam linacs despite the differential technological complexity of the system. Year-to-year beam, CIG, and PPS QA metrics show the system is well-capable of maintaining a tight tolerance on those metrics during the 5-year operation. What is most revealing in the clinical utilization data is that despite the limited range of CIG motion dictated by the system's compact design, we used only a fraction of the available angles within that range. We show in **Figure 2** that cardinal angles were the most widely used at 42.2% and 75.7% of all beams for the CIG and PPS, respectively. Our findings are like Yan et al [15] where they show that cardinal angles were mostly used in a full-gantry system. The significant reliance on the PPS cardinal angles 180° and 0° (56.9% combined) as seen in **Figure 2** is simply attributed to the fact that several disease sites, such as prostate and breast, require lateral-type beams. To achieve this, the PPS would need to be at either a180° or 0° position. By comparison, a full-gantry system plane of rotation is perpendicular to the patient medial plane at setup position, which would allow for coplanar delivery without the need for PPS cardinal-type angular positions. It is possible that some cost-savings in future designs could be realized by eliminating the need for the least usable CIG or PPS angles. Any beam projections that use PPS or CIG angles beyond what is illustrated in **Figure 1** can likely be re-planned or substituted for by alternative angle combinations without sacrificing plan quality or robustness.

The histogram data on beam range shows that there is clearly a distinct distribution for deep seated tumors (prostates) with almost all other tumors treated by beams with a range of 23 gm/cm^2^ or less. The approximate beam energy needed for a 23-gm/cm^2^ depth is 185 MeV compared with 225 MeV for a 32-gm/cm^2^ range depth. While this difference in beam energy seems modest, it may translate to a significant cost reduction in accelerator manufacturing if the user's clinical needs exclude prostate treatments. This is true for compact and full-gantry systems with potentially more savings realized in a full-gantry system because beam range is adjusted remotely and upstream in the beam line. In addition, significant vault shielding savings can be realized for both systems because less concrete wall thickness is needed to attenuate the neutrons from a lower energy accelerator.

One of the cost-saving measures in the compact system described here is the fact that there are no beam elements (quadrupole and dipole magnets) between the accelerator port and nozzle, which reduces the SAD and overall room size. However, the lack of these elements in compact systems may affect the sharpness of the distal penumbra compared with a full-gantry system. It is worth mentioning that there are additional cost savings to be realized in a compact system compared with a full-gantry system due to lower power consumption related to energizing beamline elements and driving a smaller gantry structure compared with a full-gantry system.

The nozzle structure in a full-gantry system is typically larger than the compact system described in this work because the source axis distance is larger with additional hardware components, such as an x-ray source to provide beam's-eye-view imaging. Therefore, in principle, a smaller snout allows for more beam projection choices around the patient and a sharper beam profile at depth. Our findings indicate that our air gaps are on average 10 cm smaller than full-gantry rooms [16]. This may prove consequential, especially in treating complex shaped intracranial tumors where more angle choices are typically desired to avoid involved organs at risk. Unfortunately, the smaller snout size dictates smaller field size at isocenter compared with a full-gantry snout. This could be disadvantageous for large target volumes, such as comprehensive breast and craniospinal irradiation. However, this shortcoming is mitigated through multiple isocenters and matching techniques in a compact system. Based on our patient mix, the percentage of all patients that required matching techniques was only 13.3%.

Planar imaging remains the gold standard for daily alignment in full-gantry and compact systems alike. In addition, full-gantry systems have cone-beam (CB) CT capability due to their gantry-mounted orthogonal kilovolt-to-kilovolt imagers. Compact systems like the one described in this work can be retrofitted with a separate set of non-gantry-mounted imagers to perform CBCT. While CBCT offers superior localization capabilities compared to orthogonal planar imaging, the limited field of view of CBCTs can potentially be a challenge for adaptive purposes compared with in-room, big-bore CTs, such as the AIRO system. However, the use of in-room CTs remain limited in large and compact proton centers alike due to cost and space limitations for stowing and maneuvering the CT inside the room.

One of the limitations of this study is a lack of operational comparison to compact PBS systems. Many of QA metrics that were tracked and reported in this work have similarities to PBS systems. For example, mechanical and imaging isocenter congruency and machine output. Other aspects of delivery for PBS compact systems are different that passive scatter systems. For example, the use of range shifters and layer-by-layer spot scanning may have a different impact on operational workflow that has no equivalence in passive scattering systems. Uptime can be defined similarly in addition to disease sites statistics and choice of beams and air gaps. Therefore, this study highlights some major quantifiable metrics that can be tracked in any platform (PBS or passive scatter) for objective comparisons of operational performance.

The data presented in this work could potentially guide future compact system designs so that the least-used functionalities can be phased out over time with the goal of lowering the overall system cost while maintaining its clinical robustness. It can also serve as a reference for baseline recommendations on QA schedules and tolerances for various components. We hope that similar work be replicated in the future on different commercially available PT systems with the goal of making compact systems cost effective and efficient. The economics of compact systems has the potential to make PT a modality accessible to both smaller community hospitals and larger academic centers.
